# The Natural Growth of Subsolid Nodules Predicted by Quantitative Initial CT Features: A Systematic Review

**DOI:** 10.3389/fonc.2020.00318

**Published:** 2020-03-27

**Authors:** Chen Gao, Jiaying Li, Linyu Wu, Dexing Kong, Maosheng Xu, Changyu Zhou

**Affiliations:** ^1^The First Clinical Medical College of Zhejiang Chinese Medical University, Hangzhou, China; ^2^Department of Radiology, The First Affiliated Hospital of Zhejiang Chinese Medical University, Hangzhou, China; ^3^School of Mathematical Sciences, Zhejiang University, Hangzhou, China

**Keywords:** subsolid nodule, ground glass nodule, natural growth, quantitative, CT features, systematic review

## Abstract

**Background:** The detection rate for pulmonary nodules, particularly subsolid nodules (SSNs), has been significantly improved. The purpose of this review is to summarize the relationship between quantitative features of initial CT imaging and the subsequent natural growth of SSNs to explore potential reasons for these findings.

**Methods:** Relevant studies were collected from a literature search of PubMed, Embase, Web of Science, and Cochrane. Data extraction was performed on the patients' basic information, CT methods, and acquisition methods, including quantitative CT features, and statistical methods.

**Results:** A total of 10 relevant articles were included in our review, which included 850 patients with 1,026 SSNs. Overall, the results were variable, and the key findings were as follows. Seven studies looked at the relationship between the diameter and growth of SSNs, showing that SSNs with larger diameters were associated with increased growth. An additional three studies which focused on the relationship between CT attenuation and the growth of SSNs showed that SSNs with a high CT attenuation were associated with increased growth.

**Conclusion:** CT attenuation may be useful in predicting the natural growth of SSNs, and mean CT attenuation may be more useful in predicting the natural growth of pure ground glass nodules (GGNs) than part-solid GGNs. While evaluation by diameter did have some limitations, it demonstrates value in predicting the growth of SSNs.

## Introduction

Lung cancer is one of the most common malignancies in the world, with a high mortality rate (1). Thanks to the advent of low-dose CT scanning, early detection of lung cancer has become increasingly accurate and the detection rate of pulmonary nodules has been significantly improved, particularly the subsolid nodules (SSNs) (2, 3).

The SSN is defined as a hazy, hyperdense nodule on lung windows without obscurity to bronchovesicular structures (4), which includes pure ground glass nodules (GGNs) and part-solid GGNs. The pathophysiology of SSNs is derived from thickening of the alveolar walls and septal interstitium or accumulation of fluid, cells, or amorphous material in the alveoli itself (5). Many lesions can be defined as SSNs, such as benign and malignant tumors, inflammatory lesions, and interstitial lung disease. However, persistent SSNs mainly consist of atypical adenomatous hyperplasia (AAH), adenocarcinoma *in situ* (AIS), minimally invasive adenocarcinoma (MIA), and invasive adenocarcinoma (IAC). These lesions are difficult to distinguish in CT imaging (6, 7). Moreover, their malignant transformation rate from preinvasive lesions to invasive lesions may reach up to 34%. Yet the potential biological basis for this transformation is not fully understood (8). The performance of current CT imaging modalities is also insufficient to differentiate these lesions. Additionally, most SSNs are small with low metabolic rates, leading to a high false-negative rate using PET/CT (9).

**Figure 1:**
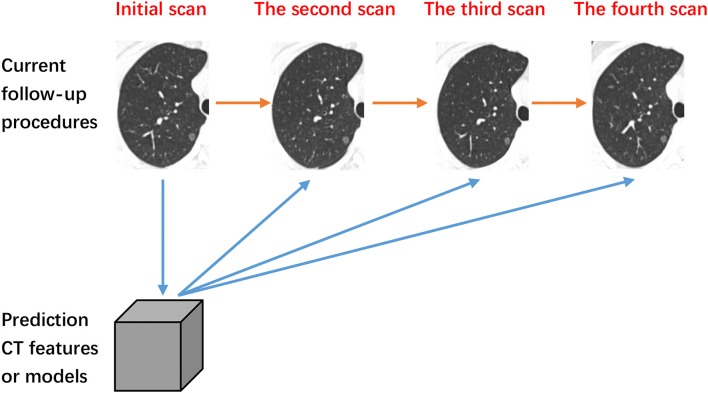
The process of follow-up. The orange line denotes current follow-up procedures. The blue lines denote our methodology of predicating the change of subsolid nodules based on initial quantitative CT features.

In predicting the course of lung disease, the growth of SSNs is commonly used as one of the key prognostic indicators for disease severity. Moreover, in 2017, the Fleischner Society released new guidelines for management of SSNs, suggesting that most nodules do require subsequent follow-up (10). While radiological observation of SSNs in follow-up CT examinations is easily doable, the changes of SSNs occur on a slow timescale, making it difficult to ascertain any discernible difference between short follow-up periods (11). This daunting waiting game is a significant cause of stress and anxiety for patients and their families. Therefore, the purpose of this review is to summarize the relationship between the quantitative features of SSN CT imaging with the future growth of SSNs. Furthermore, we seek to explore potential diagnostic indicators which can accurately predict the course and history of SSNs for further study (Figure 1).

## Methods

We conducted systematic literature searches using PubMed, Embase, Web of Science, and Cochrane from inception to October 2018. Search strategies are shown in the Supplemental Materials. No year and language restrictions were applied. These reference literatures from the selected articles were also included in additional studies to review, which were relevant to our topic. The protocol of this review was registered on PROSPERO (http://www.crd.york.ac.uk/PROSPERO; CRD42019124138).

After duplicates were eliminated, articles were selected which met the following criteria based on their title and abstract: (1) relate to the SSNs; (2) relate to the growth of primary lung cancers, lung nodules, or SSNs; and (3) study quantitative CT features associated with growth of SSNs. Some studies were excluded, including artificial nodule studies, those undergoing intervention before follow-up or during the follow-up, animal research studies, reviews, case reports, conference abstracts, comments, editorials, letters, and guidelines. The selected articles were then analyzed in full text. The title and abstract of studies retrieved using the search strategy and those from additional sources will be screened independently by two review authors to identify studies that potentially meet the inclusion criteria outlined above. The full text of these potentially eligible studies will be retrieved and independently assessed for eligibility by two review team members. Any disagreement between them over the eligibility of particular studies will be resolved through discussion with a third reviewer.

Data analysis included the patient population, year, data inclusion criteria, the number of nodules, types of nodules, CT scanner, reconstruction algorithm, reconstruction slice thickness, respiratory control, acquisition of CT quantitative features, statistical models, outcome features, assessment of SSNs, CT quantitative features, results, and key findings. Two team members independently extracted and organized data, and the remaining team members were consulted in order to resolve discrepancies in the data synthesis process.

## Results

**Figure 2:**
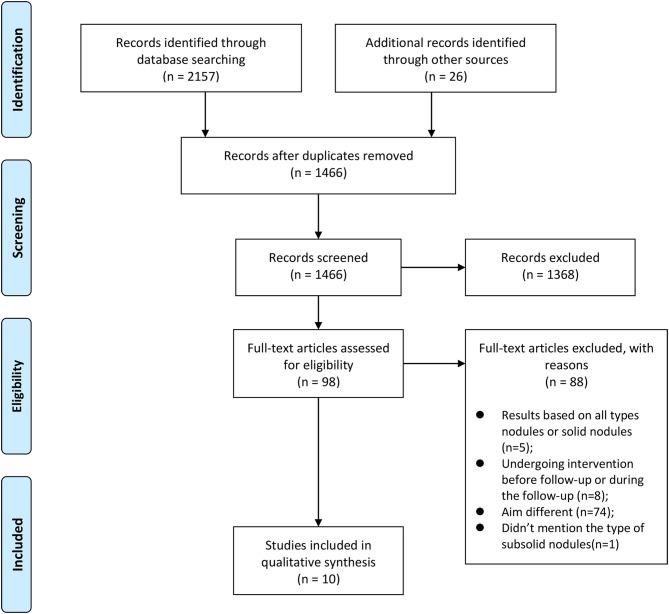
The process of our search strategy.

After searching databases using the aforementioned strategy, a total of 2,157 records were found. Twenty-six studies from the reference of 2,157 selected articles were also included in additional studies. Then, 717 duplicate references were excluded, and 1,466 articles were analyzed using their titles and abstracts. The process was described in Figure 2. A total of 98 studies were examined in full text. Finally, 10 relevant articles were included in this review (Tables 1,2).

**Table 1:** Initial patient characteristics and CT methods.

**Study**	**Patients/nodules**	**Sex (M/F)**	**Type**	**Scanners**	**Reconstruction algorithm^#^**	**Thickness (mm)**	**Acquisition method of quantitative CT features**
Bak et al. (12)	49/54	26/23	54 PG	1	Bone	2–2.5	Manual ROI segmentation and automatic extraction of data
Tamura et al. (13)	53/63	23/40^*^	63 PG	1	NM	2	Direct measurement
Eguchi et al. (14)	124/124	37/87	124 PG	3	NM	1.25	Size by direct measurement; CT attenuation by manual ROI segmentation and automatic extraction
Chang et al. (15)	89/122	73/16	122 PG	2	HSF**^+^**	1; 5	NM
Borghesi et al. (16)	19/22	12/7	7 PG; 15 PSN	2	Sharp	1	Semiautomatic ROI segmentation and automatic extraction of data
Oda et al. (17)	39/47	13/26	28 PG; 19 PSN	2	Bone plus^&^	1; 1.25; 5	Semiautomatic ROI segmentation and automatic extraction of data
Hiramatsu et al. (18)	125/125	51/74	95 PG; 30 PSN	1	NM	1.25; 2	Direct measurement
Lee et al. (19)	114/175	42/72	143 PG; 32 PSN	3	NM	1; 3	NM
Kobayashi et al. (20)	67/120	22/45	91 PG; 29 PSN	2	NM	2; 5	Direct measurement
Matsuguma et al. (21)	171/174	71/103^*^	98 PG; 76 PSN	2	NM	0.5; 1	Direct measurement

*F, female; HSF, high spatial frequency; M, male; PSN, part-solid ground glass nodules; NM, not mentioned; PG, pure ground glass nodules; ROI, region of interest*.

* *The ratio of sex was based on the number of nodules*.

# *The reconstruction algorithm was used to reconstruct the lung window*.

+ *The high-spatial-frequency algorithm was used for a 64-detector row scanner, but the other scanner was not mentioned*.

& *The bone plus algorithm was used for thin-slice helical scans, but the other scanner was not mentioned*.

**Table 2:** Information of SSNs and results.

**Study**	**Size of baseline SSNs (mm) ± SD**	**Definition of growth**	**Assessment types of SSNs**	**Including quantitative initial CT features**	**Statistically significant results**
Bak et al. (12)	11.7 ± 5.4	DE	VA	Diameter of GGO component; volume; mass; density; histogram	Histogram
Tamura et al. (13)	11.4 ± 4.2	DE	VA	Diameter; mean CT attenuation	Mean CT attenuation
Eguchi et al. (14)	7.4 ± 2.8	DE	VA	Diameter; mean CT attenuation	Diameter; mean CT attenuation
Chang et al. (15)	5.5	D	TDR	Diameter	Diameter
Borghesi et al. (16)	16.5	VDT; MDT	VA	Diameter; volume; mean CT attenuation; mass	None
Oda et al. (17)	13.0 ± 4.9	VDT	VA	Diameter; mean CT attenuation	Mean CT attenuation
Hiramatsu et al. (18)	8.3	DSE	VA	Diameter; mean CT attenuation	Diameter
Lee et al. (19)	7.8 ± 4.4	D	VA	Diameter	Diameter
Kobayashi et al. (20)	9.0	D	VA	Diameter	Diameter
Matsuguma et al. (21)	NM	DSE	VA	Diameter	Diameter

*D, an increase in diameter ≥2 mm; DE, an increase in diameter ≥2 mm or an emerging solid portion; DSE, an increase in diameter or the size of the solid part ≥2 mm or an emerging solid portion; SSN, subsolid nodule; GGO, ground glass opacity; MDT, mass doubling time; NM, not mentioned; TDR, tumor shadow disappearance rate; VA, visual assessment; VDT, volume doubling time*.

### Subjects

For these 10 studies, a total of 850 patients with 1,026 SSNs were recruited. Eight of ten studies examined a sex ratio of patients, and the other two (13, 21) examined a sex ratio of nodules (Table 1). The number of nodules was equal to the number of patients in two of the studies (14, 18). In the study conducted by Kobayashi et al. (20), the number of nodules is 120, which is about twice the number of patients. Four studies (12–15) only included pure GGNs, and the other six studies (16–21) included both pure GGNs and part-solid GGNs. The results of five studies (16–20) were based on all types of SSNs, while one study (21) was based on each type of SSNs.

### CT Parameters

Three studies (12, 13, 18) used a single CT scan, five studies (15–17, 20, 21) used two different CT scans, and two studies (14, 19) used three different CT scans (Table 1). Only one study (12) showed all reconstruction algorithms using a bone algorithm. Three studies (13, 14, 16) only used a single reconstruction thickness, which was 2, 1.25, and 1 mm, for the respective studies. Three studies (15, 16, 18) conducted CT scans at the end inspiration, and one study (13) conducted the CT scan at mid-inspiration during one breath hold.

### Classification of SSNs

One study (15) defined pure ground glass opacities (GGOs) or mixed GGO based on the tumor shadow disappearance rate (TDR). Nine studies (12–14, 16–21) used visual assessments in the definitions of SSNs (Table 2).

### Definitions of SSN Changes

Three studies (15, 19, 20) defined a change in SSNs as an increase in diameter ≥2 mm. Three different studies (12–14) defined a change in SSNs using the parameter above and/or the presence of an emerging solid portion. Two more studies (18, 21) defined a change in SSNs as the two parameters mentioned above and/or an increase in the solid parts' size. Two studies (16, 17) used volume doubling time (VDT) and/or mass doubling time (MDT) to define changes of SSNs.

### Features of Quantitative CT Imaging

#### Acquisition

Three studies (12, 16, 17) first used a segmented region of interest (ROI), followed by extraction of quantitative CT features (Table 1). Four studies (13, 18, 20, 21) acquired quantitative features using manual measurement without segmentation of ROI. Two studies (15, 19) did not mention their acquisition methods.

#### Diameter

All 10 articles studied the relationship between diameter and the growth or growth rate of SSNs. Four out of the 10 articles only included diameter as their quantitative feature (Table 2). One article (12) used the diameter of the GGO component and the diameter of the solid component in place of the diameter of the whole SSNs. The results of six studies (14, 15, 18–21) showed that SSNs with large diameters were associated with increased growth. On the contrary, two studies (12, 13) pointed to no significant relationship between the diameter of SSNs and their growth, and the results of two other studies (16, 17) showed that there was no significant relationship between the diameter of SSNs and their growth rate.

#### CT Attenuation

Six articles (12–14, 16–18) studied the relationship between CT attenuation and the growth or growth rate of SSNs (Table 2). Five articles (13, 14, 16–18) used mean CT attenuation as their measure. However, one study (12) solely relied on the histogram of CT attenuation without mean CT attenuation. The results of two articles (13, 14) showed that SSNs with large mean CT attenuation was associated with increased growth. The results of study 17 showed that SSNs with large mean CT attenuation were associated with low VDT. However, study 18 showed no significant relationship between the mean CT attenuation of initial SSNs and their subsequent growth. Study 16 showed no significant relationship between the mean CT attenuation of initial SSNs and their growth rate.

#### Volume

Two studies (12, 16) used volume as a signature to predict the growth or growth rate of SSNs (Table 2). Studies 12 and 16 pointed to no significant relationship between the initial volume and growth or growth rate of SSNs.

#### Mass

Two articles (12, 16) studied the relationship between mass and the growth or growth rate of SSNs and indicated no significant relationship (Table 2).

### Results of Different Types of SSNs

In studies (16–20) in which the results were based on all types of SSNs (Table 2), three articles (18–20) studied the relationship between the diameter and the growth of SSNs. The results of three articles (18–20) showed significant differences in diameter between rapidly growing and non-growing SSNs. And in studies (12–15, 21) in which the results were based on pure GGNs (Table 2), all articles studied the relationship between diameter and the growth of pure GGNs. However, only three articles (14, 15, 21) showed significant differences in diameter between rapidly growing and non-growing pure GGNs.

In studies (12–14) whose results were based on pure GGNs (Table 2), all articles showed significant differences in CT attenuation between rapidly growing and non-growing GGNs.

## Discussion

In this study, we reviewed the relationship between quantitative features of initial CT imaging and the growth of SSNs. Quantitative features, such as diameter, CT attenuation, volume, and mass, were investigated in 10 articles. The majority of these articles pointed to at least one significant correlation between a quantitative feature and the growth of SSNs.

In five studies (16–20), three articles (18–20) studied the relationship between diameter and the growth of SSNs, and two articles (16, 17) studied the relationship between diameter and the growth rate of SSNs. The results of three articles (18–20) all showed significant differences in diameter between rapidly growing and non-growing SSNs. However, the results of the other two articles (16, 17) showed there is no significant correlation between diameter and VDT. In another five studies (12–15, 21), all articles noted the relationship between diameter and the growth of pure GGNs. However, only three of these articles (14, 15, 21) showed there were significant differences in diameter between rapidly growing and non-growing pure GGNs. The reason is as follows: firstly, as a two-dimensional parameter, the diameter does have some limitations. For example, most nodules are asymmetrical (22), making diameter an inaccurate representation of the entire SSNs. Secondly, this may be attributable to the small sample size and/or lead time bias from varying lengths of follow-up (23, 24). VDT on the other hand included information about the change of volume and the length of follow-up. Many studies used VDT to estimate the growth of a nodule, to classify tumors, and to evaluate malignancy (17, 25). Moreover, the growth pattern of malignant nodules is exponential (26), and SSNs with large diameters will grow faster than their smaller counterparts. Thus, we believe that measurement parameters such as diameter or volume will have value in predicting the growth of SSNs.

Most AISs/MIAs were displayed as GGO, and IACs were also found to be solid nodules (27–30). CT attenuation of GGNs was related with the physical cell density (31). The results of Barletta et al. (32) suggested that the solid pattern of SSNs was associated with cytologic atypia and tumor growth. Following this train of thought, SSNs with greater CT attenuation are more likely to be rapidly growing or malignant. And the results of three selected articles supported this view (12–14). In these studies (12–14) in which the results were based on pure GGNs, all articles showed significant differences in CT attenuation between rapidly growing and non-growing SSNs. However, two studies (13, 14) included mean CT attenuation into their analyses, and one study (12) included a histogram of CT attenuation. The mean CT attenuation only represented the overall density of nodules while other indicators, such as max CT attenuation, standard deviation CT attenuation, and a histogram of CT attenuation, represented information about the heterogeneity of SSNs. It was found that SSNs with a high degree of heterogeneity was malignant and invasive (33–35). Therefore, SSNs with large mean CT attenuations and great heterogeneity will grow even more rapidly, indicating a poor prognosis. Thus, mean CT attenuation, max CT attenuation, standard deviation of CT attenuation, and a histogram of CT attenuation may be useful in predicting the growth of SSNs.

Interestingly, in two studies (16, 17) in which the results were based on all types of SSNs, the two articles also studied the relationship between mean CT attenuation and the growth rate of SSNs. Only one study (17) showed that SSNs with greater CT attenuation are more likely to be rapidly growing. This may be the cause in the different rates of two types of SSNs. In the study (16), they included seven pure GGNs and 15 part-solid GGNs. And in another study (17), they included 28 pure GGNs and 19 part-solid GGNs. In study (16), they mentioned that some part-solid GGNs showed a reduction in mean CT attenuation during the follow-up. If the growth of the GGO component in part-solid GGNs is greater than the growth of the solid component, it might reduce mean CT attenuation. Thus, mean CT attenuation may be more useful in predicting the growth of pure GGNs than part-solid GGNs.

There are several limitations to the current study. Firstly, few cases were included in selected studies. The small sample size negatively impacts the credibility of those studies (23, 24). Among the reasons may be that inclusion criteria are strict and follow-up is longer. Another reason may be that patients with SSNs are simply too anxious to endure long periods of follow-up and will request biopsy or surgery for definitive diagnosis. Another limitation is regarding the variable inclusion criteria among articles. Some articles only included SSNs with confirmed pathology while some also included patients with a history of cancer. Thirdly, the length of follow-up was variable. If the follow-up time is insufficient, the growth of SSNs may be attributed to a measurement error. Fourthly, studies used different reconstruction methods or scanning machines, which may affect the value of measured CT features (36). Lastly, while these quantitative CT imaging features may predict the growth of SSNs, the underlying causes and natural history of SSNs have not yet been fully understood.

Several challenges are worth noting. The segmentation of SSNs was used in most studies, including manual, semiautomatic, and automatic segmentation. At present, manual segmentation is considered the gold standard. However, manual segmentation is time-consuming and suffers from interobserver variation (37, 38). Physicians pay more attention to semiautomatic segmentation as the new standard. LungCARE software, for instance, uses two algorithms (sharp, B60F; medium sharp, B50F), to segment pure GGNs. The ratios of successful segmentation were a staggering 98.3 and 97.8% with sharp and medium sharp reconstruction algorithms, respectively (39). However, the boundary between SSNs and the surrounding lung parenchyma is hazy, and the size and CT attenuation of SSNs are susceptible to changes in respiration (40, 41). Thus, the performance of said software models needs to be verified in further studies, and the segmentation technology definitely could benefit from further development. Secondly, quantitative CT features such as diameter, volume, and CT attenuation are common and visible. With the development of computer technology, many radiologists attach importance to the radiomic features which are quantitatively extracted from medical imaging, such as the gray-level co-occurrence matrix (GLCM) and run-length matrix (RLM) (42). A study by Wang et al. (43) retrospectively illustrated the potential predictive power of quantitative features to differentiate malignant from benign lung nodules. One hundred fifty radiomic features were extracted from 593 patients' CT imaging and shown to poise predictive power using a support vector machine. For their training group and testing group, the sensitivity and specificity of diagnosis were 82.5% sens. (165 of 200), 89.5% spec. (179 of 200), and 74.6% sens. (91 of 122), 78.9% spec. (56 of 71), respectively. Radiomic features may be of value in improving the predictive model and growth model of SSNs.

## Conclusion

This study examined the predictive power of quantitative CT imaging features for predicting the growth of SSNs across several studies. While using the diameter of SSNs as a prognostic indicator did have some limitation, it demonstrated value in predicting the growth of SSNs. CT attenuation may be useful in predicting the growth of SSNs, and mean CT attenuation may be more useful in predicting the growth of pure GGNs than part-solid GGNs. Accurate and timely detection of SSNs and their plausible growth can offer great value in directing appropriate treatment options and lowering patient anxiety. Further studies are needed to explore the predictive potential of quantitative CT imaging and radiomic features to understand the natural history of SSNs.

## Data Availability Statement

The datasets analyzed in this article are not publicly available. Requests to access the datasets should be directed to Maosheng Xu, xums166@zcmu.edu.cn.

## Author Contributions

CG: manuscript draft, search strategy, literature review, study selection, data extraction, review of final draft. JL: literature review, study selection, data extraction. LW: literature review, study selection, data extraction. DK: review of final draft. CZ: conception and design of the study, search strategy, review of final draft. MX: conception and design of the study, search strategy, and review of final draft.

### Conflict of Interest

The authors declare that the research was conducted in the absence of any commercial or financial relationships that could be construed as a potential conflict of interest.
