# Supplementary material for: The Natural Growth of Subsolid Nodules Predicted by Quantitative Initial CT Features: A Systematic Review
Source: Front Oncol. 2020 Mar 27;10:318. doi: 10.3389/fonc.2020.00318 (PMC7119340; doi:10.3389/fonc.2020.00318)
Supplement: Supplementary file 1 [file Data_Sheet_1.docx]

**Supplementary Material**

Search Strategy of four database

PubMed


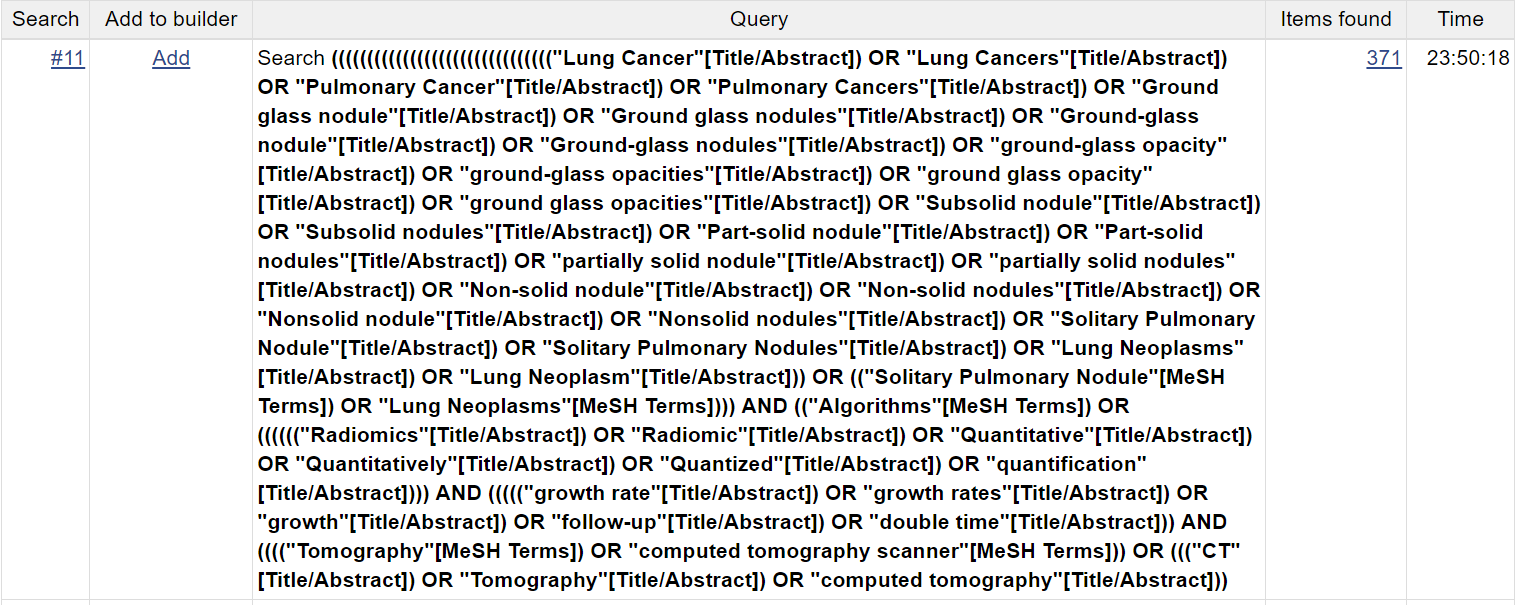


Web of Science


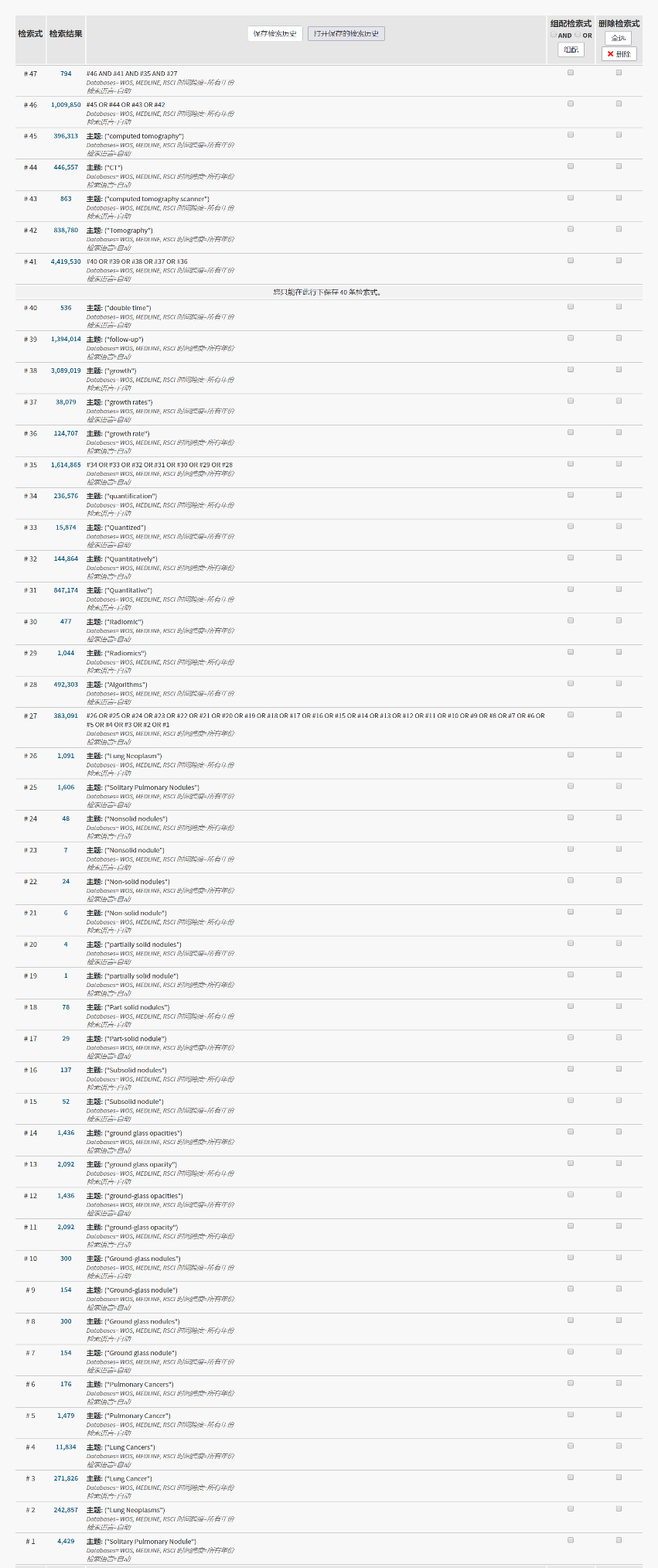


Embase
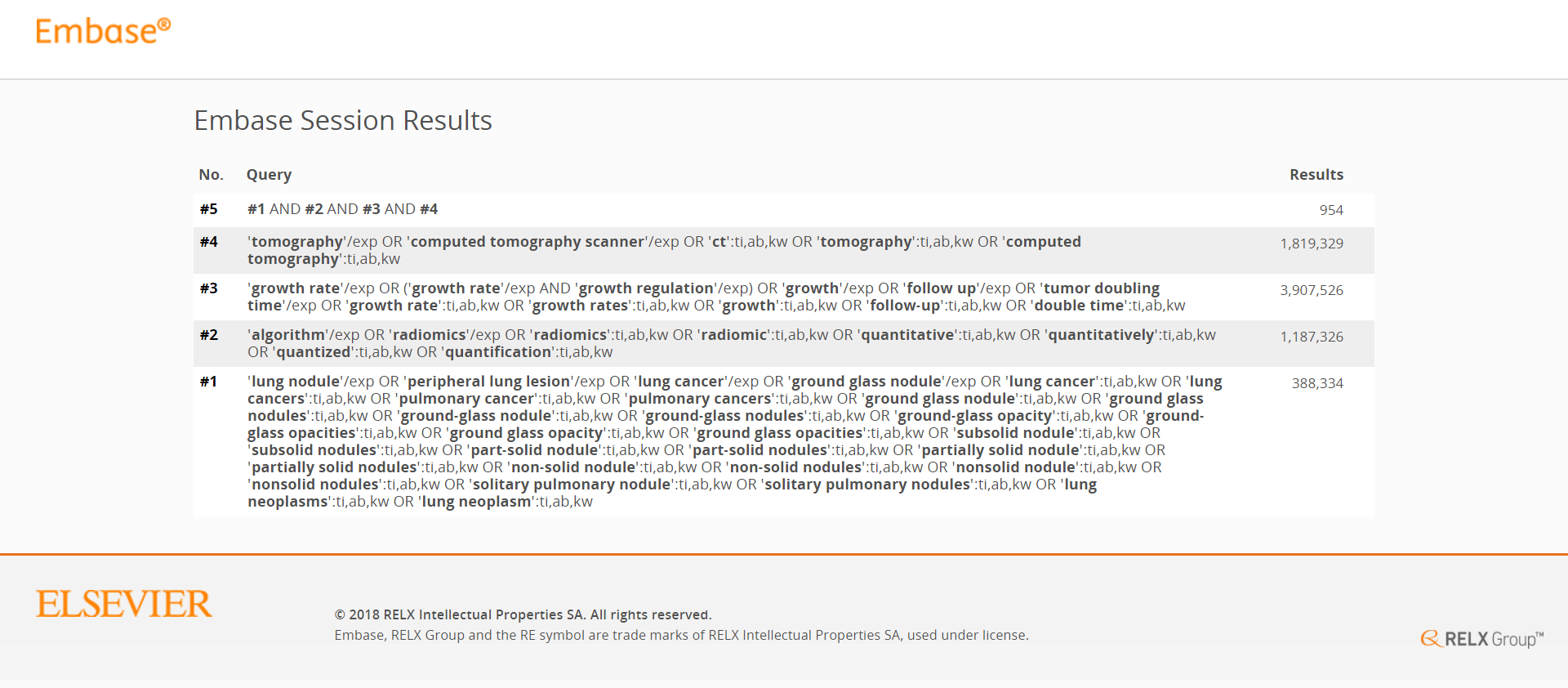


Cochrane Library


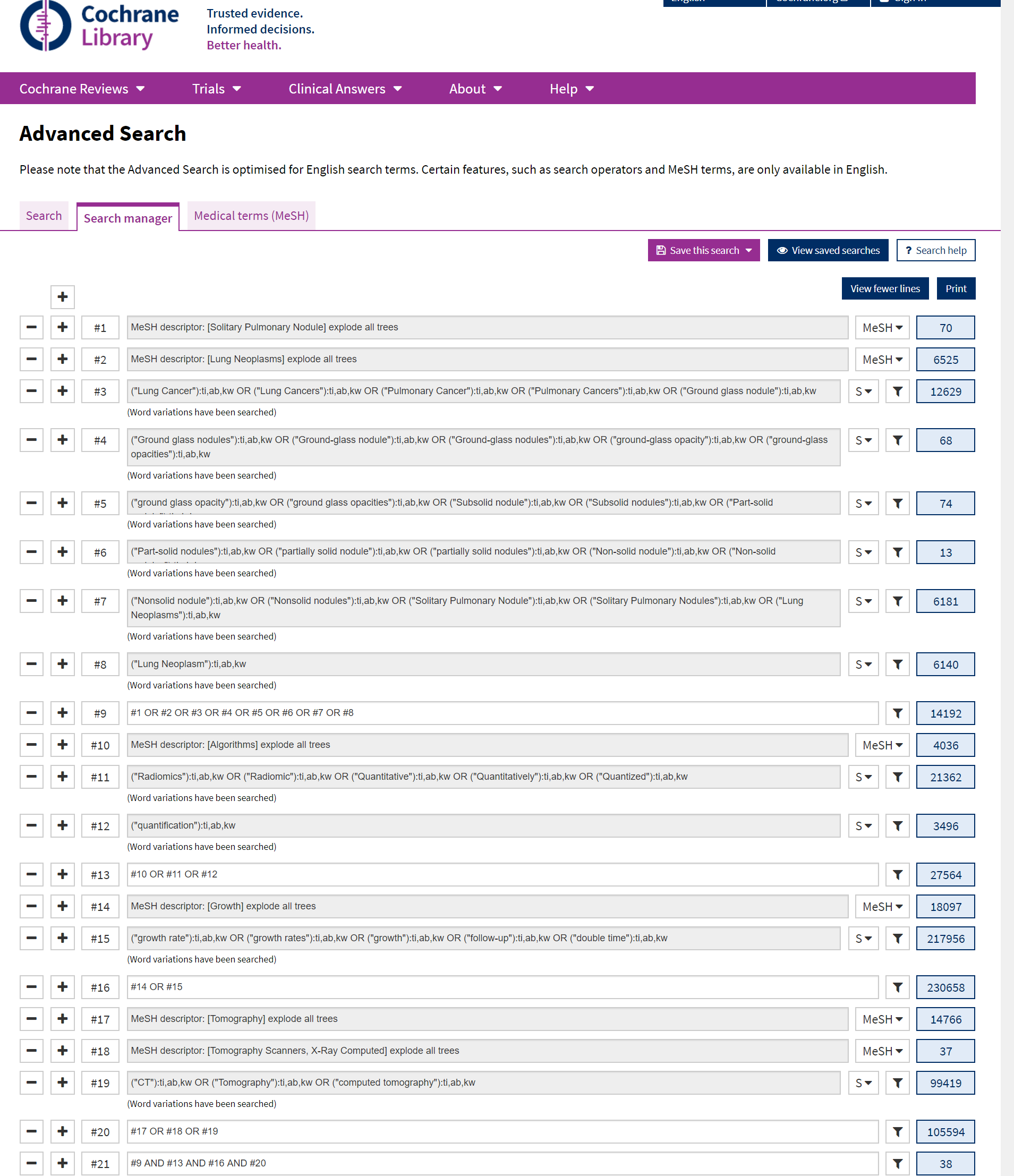


| Study | Information of CT scanning follow-up |
| --- | --- |
| Bak SH et al. ^[12]^ | 24.2 ±16.9 months (range, 2.2-64.9 months) |
| Tamura M et al. ^[13]^ | 26.1±4.6months (Mean ± SD) |
| Eguchi T et al. ^[14]^ | 57 months (range, 24.1-113.6 months) |
| Chang B et al. ^[15]^ | 59 months (range, 25-140 months) |
| Borghesi A et al. ^[16]^ | 573 days (range, 126–1128 days) |
| Oda S et al. ^[17]^ | 188 days (range, 34–1486 days) |
| Hiramatsu M et al. ^[18]^ | 1048 days (range, 177-3269 days) |
| Lee SW et al. ^[19]^ | 48 months (range, 24-99 months) |
| Kobayashi Y et al. ^[20]^ | 4.2 years (range,0.5–12.0 years) |
| Matsuguma H et al. ^[21]^ | 29 months (range, 1-136 months) |

**Supplement Table**. Information of CT scanning follow-up
